# Oxidative allylic rearrangement of cycloalkenols: Formal total synthesis of enantiomerically pure trisporic acid B

**DOI:** 10.3762/bjoc.7.54

**Published:** 2011-04-11

**Authors:** Silke Dubberke, Muhammad Abbas, Bernhard Westermann

**Affiliations:** 1 Leibniz Institute of Plant Biochemistry, Department of Bioorganic Chemistry, Weinberg 3, D-06120 Halle (Saale), Germany; 2 Chair of Advanced Proteomics and Cytomics Research, Faculty of Science, Department of Zoology, King Saud University, P.O. Box 2455, 11415 Riyadh, Saudi Arabia; 3 Martin-Luther-University, Department of Organic Chemistry, Kurt-Mothes-Str. 2, D-06120 Halle (Saale), Germany

**Keywords:** enzymatic resolution, natural products, oxidative rearrangement, pig liver esterase (PLE), trisporic acid B

## Abstract

Enantiomerically highly enriched unsaturated β-ketoesters bearing a quaternary stereocenter can be utilized as building blocks for the synthesis of natural occurring terpenes, i. a., trisporic acid and its derivatives. An advanced building block has been synthesized in a short reaction sequence, which involves an oxidative allylic rearrangement initiated by pyridinium dichromate (PDC) as the key step.

## Introduction

**Scheme 1:**
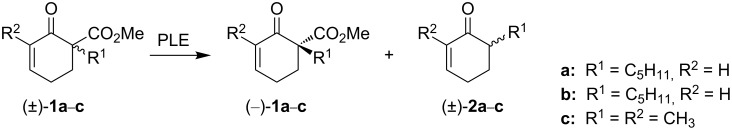
PLE (pig liver esterase)-catalyzed saponification of β-ketoesters **1**.

The generation of chiral, non-racemic compounds bearing a stereogenic quaternary carbon centre is of great interest [1–8]. Therefore, much effort has been directed towards the synthesis of this stereogenic unit and solutions have been found, e.g., by Christoffers and d’Angelo [9–11]. We have already disclosed our results to obtain these products highly enantiomerically enriched by pig liver esterase (PLE) catalyzed saponification of α-substituted β-ketoesters [12–13]. We have now extended this methodology towards the saponification of unsaturated β-ketoesters (±)-**1a**,**b** (Scheme 1) to provide a convenient access to highly functionalized cyclohexenones in optically pure form. In addition, we show that non-racemic chiral α-substituted, β-ketoesters such as (−)-**1c** can be easily obtained by the enzyme-catalyzed hydrolysis of the ester moiety.

**Figure 1:**
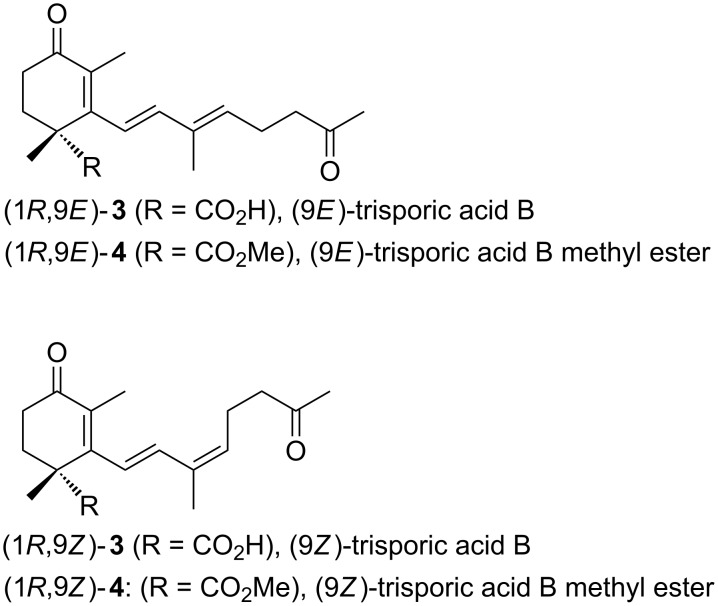
(9*E*)- and (9*Z*)-trisporic acid B.

The β-ketoesters **1** are of particular interest because they can be envisioned as suitable starting materials for natural product syntheses and have important technical applications. For example, enantiomerically pure **1a** can be used as building block for the synthesis of dienes leading to nagilactones, **1b** has been utilized for the preparation of ferroelectric liquid crystals [14–15]. Starting from (−)-**1c** a vast number of cyclohexene carboxylic acid-based natural products bearing additional alkyl substituents can be synthesized. These structural features can be found in the cyclic framework of (9*E*)- and (9*Z*)-trisporic acid B ((9*E*)-**3**, (9Z)-**3**) and its methyl esters (9*E*)-**4** and (9*Z*)-**4**, which are very potent fungal pheromones [16–20]. Both the 9*E* and the 9*Z* isomer occur naturally (Figure 1).

Trisporic acids have been isolated from cultures of mucoraceous fungi: *Blakeslea trispora, Mucor mucedo* and *Phycomyces blakesleeanus* [16]. They stimulate caratenogenesis and zygophore (sex cell) formation. Interestingly, for the biosynthesis of the trisporic acid, mating species of the opposite sex have to cooperate. It has been suggested, that these carotenoid-derived substances both regulate and stimulate the first stages of sexual development [17,19]. In this publication, the formal total synthesis of optically pure (9*Z*)-trisporic acid methyl ester (1*R*,9*Z*)-**4** (Figure 1) will be described, starting from the methyl (1*R*)-1,3-dimethyl-2-oxocyclohex-3-ene-1-carboxylate ((−)-**1c**, Scheme 1) [21–22].

## Results and Discussion

**Scheme 2:**
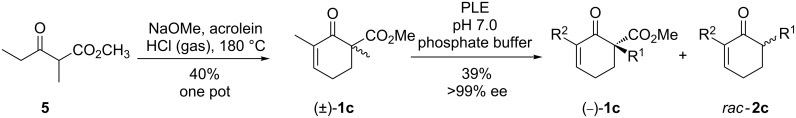
Synthesis and PLE-catalyzed saponification of β-ketoester **1c**.

Precursor for the synthesis of racemic cyclohexenone (±)-**1c** is methyl 2-methyl-3-oxopentanoate (**5**), which upon addition of acrolein undergoes a tandem Michael/aldol-reaction sequence (Scheme 2) [11]. To obtain optically pure β-ketoester **1c**, the racemate **1c** is resolved by a pig liver esterase (PLE)-catalyzed saponification reaction. As in other cases reported earlier, the racemic β-ketoesters are hydrolyzed with high stereoselectivity allowing the isolation of optically pure esters (–)-**1**. The corresponding hydrolysis products, the (+)-β-ketoacids decarboxylate and racemize to cyclohexenones **2** during the enzymatic reaction and workup (Scheme 1) [12,14]. Treatment of (±)-**1c** in phosphate buffer at pH 7.0 led to selective saponification of the (*S*)-isomer (+)-**1c** to give *rac*-**2c**.

The enantiomeric excess of (−)-**1c** was monitored during the reaction, and after completion of the reaction the enantiomeric excess was determined to be >99% by GLC using a cyclodextrin modified stationary phase (LIPODEX E). The *R*-configuration at the stereocenter was established by chemical correlation [10,14].

**Scheme 3:**
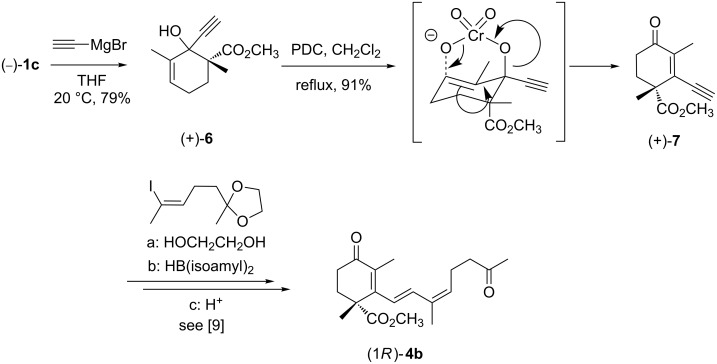
Synthesis of key building block (+)-**7**.

The first step towards the synthesis of cyclohexenone (+)-**7**, which is the key building block in our synthesis, was the incorporation of a C_2_ unit into β-ketoester (−)-**1c** (Scheme 3). This was achieved by adding ethynyl magnesium bromide in THF at room temperature. The cyclohexenol (+)-**6** can be isolated in 79% yield with a diastereomeric ratio of 96:4. However, the configuration at the newly created stereogenic centre need not be determined since it is destroyed in the next step, the PDC-catalyzed rearrangement to (+)-**7**. After 7 h, no starting material was detected by TLC, allowing the isolation of the desired cyclohexenone **7** in 91% yield. Although steric interference was believed to be a major obstacle of this process, this factor turned out to be unimportant. It is assumed, that the transition state during [3,3]-sigmatropic rearrangement is as shown in Scheme 3 [23–24], where the hydroxyl group is fixed in a pseudo-axial orientation, any other orientation would cause severe steric interaction with the 3-methyl group.

In conclusion, the key intermediate *R*-(+)-**7**, which can be utilized as an enantiomerically pure starting material in Suzuki’s elegant protocol [25] towards the synthesis of racemic (9*E*)-trisporic acid B methyl ester (9*E*)-**4**, has been synthesized enantiomerically pure in a two step procedure starting from optically pure β-ketoester (−)-**1c** in an overall yield of 65%. Furthermore, we have shown that the oxidative allylic rearrangement of cycloalkenols can be carried out easily despite a high degree of functionalization and steric interactions. Therefore, this method should be applicable additionally to the synthesis of a great number of natural products such as cassiol [26–27].

## Experimental

**General.** All air- and moisture-sensitive reactions were performed under an argon atmosphere in oven-dried glassware. All solvents were dried over standard drying agents; THF was freshly distilled over sodium prior to use. Enzymatic reactions were monitored by Methrom 702 SM Titrino titrator, chiral GLC was performed with a LIPODEX E column (12 m), provided by Macherey&Nagel, Germany. Pig liver esterase (PLE) was purchased from Sigma. Reactions were monitored by TLC on silica gel 60 F254. Column chromatography was performed on silica gel 60 (70–230 mesh, ASTM). Melting points were determined with a Gallenkamp melting point apparatus in open capillaries and are uncorrected. Optical rotations were measured in solution at 589 nm with a PerkinElmer 241 polarimeter on a 1.00 dm cell. ^1^H (200 MHz) and ^13^C (50 MHz) NMR spectra were recorded with a Bruker AMX-200 with TMS as internal reference. Coupling constants *J* are given in Hz, the carbon multiplicities were assigned by DEPT 135 pulse sequence techniques (s = singlet, d = doublet, t = triplet, q = quadruplet). IR spectra were recorded with a Nicolet 510 FT-IR spectrometer, and GC–MS analysis was performed with a Finnigan MAT Magnum System 240, Varian GC 3400 DB 5. Elemental analysis was carried out with a PerkinElmer elemental analysator 240 at the University of Paderborn, Germany.

**Methyl (−)-(1*R*)-1,3-dimethyl-2-oxocyclohex-3-ene-1-carboxylate (1c).** Methyl 2-methyl-3-oxopentanoate (**5**) (3.42 g, 24.0 mmol) was added dropwise to a solution of sodium methoxide prepared from sodium (0.02 g, 0.9 mmol) and methanol (30 mL). The reaction mixture was cooled to 0 °C (ice bath), after which freshly distilled acrolein (1.34 g, 24.0 mmol) in methanol (8 mL) was added dropwise. Stirring was continued at 20 °C for approx. 12 h. The mixture was cooled in ice, and HCl (gas) introduced until the color changed to red (approximately after 2–3 h); stirring was then continued at 20 °C for about 12 h. The reaction mixture was filtered and evaporated to dryness. After the addition of a catalytic amount of hydroquinone the dark brown residue was heated at 180 °C for 1 h and then purified by distillation under reduced pressure to give (±)-**1c** (1.70 g, 40%). The kinetic resolution was carried out as follows: To phosphate buffer (pH 7.0, 0.1 M, KH_2_PO_4_/K_2_HPO_4_, 200 mL) was added (±)-**1c** (1.50 g, 8.2 mmol) and PLE (100 µL) and the resulting mixture stirred at 20 °C. The pH was maintained constant by periodic addition of NaOH (2 N). During the reaction, the ee value was monitored by GLC (LIPODEX E). After the reaction was complete, the mixture was acidified with aqueous HCl to pH 2 and extracted overnight using a continuous liquid/liquid extractor with Et_2_O. After drying of the organic layer (MgSO_4_), the solvent was evaporated and the remaining oily residue distilled in a Kugelrohr apparatus to afford (−)-**1c** (590 mg, 39%); bp 88 °C/1.1 Torr; 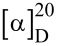 −81.2 (*c* 1.55, CHCl_3_); IR (film): 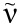 2978, 2872, 1736, 1676 cm^−1^; ^1^H NMR (CDCl_3_): δ 1.27 (s, 3H, CH_3_), 1.70 (s, 3H, CH_3_), 1.68–1.85 (m, 1H), 2.21–2.44 (m, 3H), 3.59 (s, 3H, OCH_3_), 6.58 (dd, *J* = 3.2 Hz, 1H, =CH); ^13^C NMR (CDCl_3_): δ 16.7 (q), 20.6 (q), 23.6, 33.9 (2 t), 52.6 (q), 53.5 (s), 134.9 (s, =C-), 144.5 (d, =CH), 173.6 (s, CO), 197.6 (s, CO); GC–MS (80 eV) *m*/*z* (%): 182 (25), 167 (28), 150 (50), 135 (15), 123 (41), 95 (22), 82 (100), 79 (14); Anal. Calcd for C_10_H_14_O_3_ (182.2): C, 65.92; H, 7.74. Found: C, 65.78; H, 7.75.

Methyl (+)-(1*R*,2R*)-2-ethynyl-2-hydroxy-1,3-dimethylcyclohex-3-ene-1-carboxylate (6). To a solution of (−)-**1c** (0.50 g, 2.75 mmol) in THF (5 mL), was added dropwise over a period of 30 min ethynyl magnesium bromide (0.5 M in THF; 9.05 mL, 4.53 mmol) at 20 °C. Stirring at this temperature was continued for 1 h, and then the reaction mixture was quenched by adding sat. aq NH_4_Cl-solution (3 mL). The organic layer was separated, and the aqueous phase extracted with Et_2_O (3 × 50 mL). The organic layers were combined and washed in turn with 10% HCl, brine, dried (MgSO_4_), and concentrated under reduced pressure. The residue was purified by column chromatography (silica gel, petroleum/ethyl acetate 9:1) to give colorless crystals of **6** (0.45 g, 79%); mp 79.2 °C; *R*_f_ = 0.21 (petroleum/ethyl acetate 9:1); 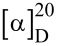 +232.4 (*c* 1.17, CHCl_3_); IR (KBr): 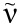 3478, 3244, 2980, 2959, 2933, 2100, 1705 cm^−1^; ^1^H NMR (CDCl_3_): δ 1.27 (s, 3H, CH_3_), 1.86 (s, 3H, CH_3_), 1.92–2.21 (m, 4H), 2.44 (s, 1H, ≡CH), 3.78 (s, 3H, CH_3_), 4.11 (s, 1H, OH), 5.43 (s, 1H, =CH); ^13^C NMR (CDCl_3_): δ 17.1 (q), 18.1 (q), 22.0 (t), 27.9 (t), 50.3 (s, C-1), 52.6 (q), 71.6 (s, C-2), 72.5 (d, ≡CH), 86.5 (s, C≡), 123.4 (d, =CH), 134.4 (s, =C), 178.2 (s, CO); GC–MS (70 eV) *m*/*z* (%): 191 (12) [M − OH]^+^, 175 (30), 147 (18), 131 (40), 107 (85), 91 (28), 79 (100); Anal. Calcd for C_12_H_16_O_3_ (208.3): C, 69.21; H, 7.74. Found: C, 69.05; H, 7.91.

**Methyl (+)-(1*R*)-2-ethynyl-1,3-dimethyl-4-oxocyclohex-2-ene-1-carboxylate (7).** PDC (0.72 g, 1.90 mmol) was added to a solution of **6** (0.20 g, 0.90 mmol) in abs CH_2_Cl_2_ (5 mL) in the presence of a catalytic amount of hydroquinone. The resulting suspension was heated under reflux in an argon atmosphere for 7 h. At the end of the reaction, ethyl acetate (1 mL) was added and the product separated from the chromium salts by passage through a short column of silica gel (petroleum/ethyl acetate 9:1) to give **7** (0.18 g, 91%) as a colorless oil. The product required storage in a freezer to prevent polymerization. *R*_f_ = 0.1 (petroleum/ethyl acetate 9:1); 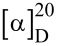 +48.4 (*c* 0.98, CHCl_3_); IR (film): 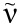 3262, 2984, 2955, 2100, 1736, 1674, 1593 cm^−1^; ^1^H NMR (CDCl_3_): δ 1.57 (s, 3H, CH_3_), 1.89–2.00 (m, 2H, CH_2_), 2.03 (s, 3H, CH_3_), 2.39–2.57 (m, 2H, CH_2_), 2.50 (s, 1H, ≡CH), 3.76 (s, 3H, OCH_3_); ^13^C NMR (CDCl_3_): δ 14.8 (q), 24.4 (q), 33.9, 34.7 (2 t), 47.5 (s), 53.0 (q), 80.6 (s, C≡), 92.5 (d, ≡CH), 138.2, 141.5 (2 s, C=C), 174.7 (s, CO), 197.4 (s, CO); GC–MS (70 eV) *m*/*z* (%): 207 (100) [M + H]^+^, 191 (50), 178 (23), 150 (21), 119 (65), 91 (50); Anal. Calcd for C_12_H_14_O_3_ (206.2): C, 69.89; H, 6.84. Found: C, 69.72; H, 7.03.
